# Barriers and Facilitators to Online Portal Use Among Patients and Caregivers in a Safety Net Health Care System: A Qualitative Study

**DOI:** 10.2196/jmir.4847

**Published:** 2015-12-03

**Authors:** Lina Tieu, Urmimala Sarkar, Dean Schillinger, James D Ralston, Neda Ratanawongsa, Rena Pasick, Courtney R Lyles

**Affiliations:** ^1^ Division of General Internal Medicine University of California, San Francisco San Francisco, CA United States; ^2^ Group Health Research Institute Seattle, WA United States; ^3^ Helen Diller Family Comprehensive Cancer Center University of California, San Francisco San Francisco, CA United States

**Keywords:** personal health records, electronic health records, chronic disease, caregivers, health literacy, safety-net providers

## Abstract

**Background:**

Patient portals have the potential to support self-management for chronic diseases and improve health outcomes. With the rapid rise in adoption of patient portals spurred by meaningful use incentives among safety net health systems (a health system or hospital providing a significant level of care to low-income, uninsured, and vulnerable populations), it is important to understand the readiness and willingness of patients and caregivers in safety net settings to access their personal health records online.

**Objective:**

To explore patient and caregiver perspectives on online patient portal use before its implementation at San Francisco General Hospital, a safety net hospital.

**Methods:**

We conducted 16 in-depth interviews with chronic disease patients and caregivers who expressed interest in using the Internet to manage their health. Discussions focused on health care experiences, technology use, and interest in using an online portal to manage health tasks. We used open coding to categorize all the barriers and facilitators to portal use, followed by a second round of coding that compared the categories to previously published findings. In secondary analyses, we also examined specific barriers among 2 subgroups: those with limited health literacy and caregivers.

**Results:**

We interviewed 11 patients and 5 caregivers. Patients were predominantly male (82%, 9/11) and African American (45%, 5/11). All patients had been diagnosed with diabetes and the majority had limited health literacy (73%, 8/11). The majority of caregivers were female (80%, 4/5), African American (60%, 3/5), caregivers of individuals with diabetes (60%, 3/5), and had adequate health literacy (60%, 3/5). A total of 88% (14/16) of participants reported interest in using the portal after viewing a prototype. Major perceived barriers included security concerns, lack of technical skills/interest, and preference for in-person communication. Facilitators to portal use included convenience, health monitoring, and improvements in patient-provider communication. Participants with limited health literacy discussed more fundamental barriers to portal use, including challenges with reading and typing, personal experience with online security breaches/viruses, and distrust of potential security measures. Caregivers expressed high interest in portal use to support their roles in interpreting health information, advocating for quality care, and managing health behaviors and medical care.

**Conclusions:**

Despite concerns about security, difficulty understanding medical information, and satisfaction with current communication processes, respondents generally expressed enthusiasm about portal use. Our findings suggest a strong need for training and support to assist vulnerable patients with portal registration and use, particularly those with limited health literacy. Efforts to encourage portal use among vulnerable patients should directly address health literacy and security/privacy issues and support access for caregivers.

## Introduction

Over the past few decades, there has been a surge in the use of electronic health records (EHRs) in the United States, spurred by the Health Information Technology for Economic and Clinical Health (HITECH) Act [1] “Meaningful Use” financial incentive program [2]. As Meaningful Use has moved into its fourth year, its incentives have promoted the rapid uptake of online patient portals by health systems nationwide [3], allowing patients to access laboratory test results, view visit summaries, and email their providers. Patient portals have been touted as a way to support self-management for chronic diseases by promoting disease awareness and knowledge, self-efficacy, and improvements in health behaviors and communication [4-9]. Early evidence also linked portal use to better outcomes such as risk factor control for diabetes [10,11].

Despite the potential of portals to promote patient engagement and improve self-management [12], there is evidence that not all patient subgroups use portals similarly. Among integrated health care systems with well-established portals, there is consistent evidence that racial/ethnic minorities and patients with lower income, education, and health literacy are significantly less likely to use available portal websites [10,13-19]. Although some studies tried to elucidate general barriers to portal use, such as computer/Internet access, attitudes/preferences, awareness, and security/privacy of information [5,7,13,20], few studies to date have reported on health literacy as a barrier to portal use and interpretation [14,21] and none have reported specific barriers among individuals with limited health literacy. Moreover, caregivers—although recognized as increasingly important in the care of those with chronic illness [22]—have often been left out of studies examining portal use to date.

A safety net hospital or health system provides a significant level of care to low-income, uninsured, and vulnerable populations. Although there have been early adopters in the field [23,24], the use of online patient portals is new territory for many safety net health systems because many have just completed implementation of their EHRs. In 2012, 40% of community clinics and health centers in California reported at least a basic EHR system [25] and even fewer had provided patients access to their personal health record information online. Given the potential for portals to improve self-management, it is important to assess the readiness and willingness of patients and caregivers to access personal health records online in safety net settings—especially because patient interest is high [26,27].

In this qualitative study, we sought to elucidate the barriers and facilitators to use of a patient portal in anticipation of portal implementation in an urban, safety net primary care clinic.

## Methods

### Research Setting

The study was conducted at San Francisco General Hospital (SFGH), a safety net hospital in the San Francisco Health Network system. From December 2013 to September 2014, we recruited participants to gain a pool of individuals with a wide range of health knowledge and engagement. Recruitment sites included (1) the General Medicine Clinic (GMC), a primary care clinic serving more than 6500 patients, most of whom are uninsured (32%) or on Medicaid (39%); (2) a diabetes support group led by diabetes nurses; and (3) a diabetes education class that GMC patients were referred to at the hospital. GMC began exclusively using an EHR in June of 2013. At the time of the interviews, the SFGH-wide patient portal was not yet launched; rollout was scheduled for early 2015.

### Sampling Procedure

We recruited patients through an electronic query of patients with upcoming clinic or diabetes group appointments. To recruit participants identified as having upcoming clinic appointments, study staff approached potential participants before or after their appointments, explained the study, and recruited interested participants. To recruit participants identified as being enrolled in group sessions, study staff attended the sessions, described the study to the group, and recruited interested participants. Caregivers, defined as someone playing a role in the management of a patient’s health other than the patient or the medical provider, were recruited by provider referral of someone who attended medical visits with or communicated with a provider on behalf of a patient. Participants were eligible for the study if they were (1) English-speaking, (2) not cognitively impaired, and (3) diagnosed with a chronic disease or the caregiver of such a patient. We focused on patients with chronic illnesses because portal use may be particularly useful in supporting ongoing self-management. We included only participants who expressed some interest in using the Internet overall to manage their health care, unless accompanied by a caregiver who expressed such interest, because we felt that this represented a realistic sample of individuals who would be potentially interested in and able to use the portal when it launched.

### Data Collection Procedure

During recruitment, we administered a short questionnaire to gather information on demographics (age, race/ethnicity, gender); diagnosis of a chronic disease (heart disease, diabetes, high blood pressure, heart failure, asthma/chronic obstructive pulmonary disease [COPD], and/or chronic kidney disease); interest in using the Internet to manage health care at SFGH (high, some, none, or don’t know/need more information); and frequency of current Internet use (daily, weekly, monthly or less, or none). Finally, we administered a previously validated one-item health literacy scale regarding how confident participants were filling out medical forms on their own (not at all, a little bit, somewhat, quite a bit, extremely) [28] because this has been shown to be predictive of portal use in our previous quantitative work [14]. We classified participants noting any lack of confidence in filling out forms as having limited health literacy.

We conducted semi-structured in-depth interviews with 11 patients and 5 caregivers, two of which were dyad interviews with both a patient and his or her respective caregiver (Multimedia Appendix 1, Interview Guide). Although the interviews included discussion of current health status, health behaviors, and health care utilization, emphasis was placed on prior use of the Internet and specific interest in the use of a patient portal website for health management, informed by the Theory of Acceptance and Use of Technology [29,30]. To provide a visual example of a patient portal interface, participants were shown screenshots of a sample patient portal interface on paper, including the log-in, test results, and visit summary features. Participants were asked to state whether they thought they would use the portal website in their own care and what features of the portal website were of most interest to them. Caregivers were asked to discuss the potential impact proxy access to a patient’s personal health records would have on their role. The interviews were transcribed and deidentified before analysis.

### Data Analysis

Using data from the questionnaires, we summarized the participant demographics and Internet and portal use responses.

Authors CL and LT read the interview transcripts in their entirety before independently analyzing them. We used an interpretive description approach [31,32] to analyze the transcripts, using inductive and deductive coding techniques. Coding was done using Atlas.ti 7 software [33]. First, we used inductive open coding to identify all emerging themes and subthemes that participants mentioned during the discussions [34,35]. To assure quality of the analysis and to uphold the constant comparison open coding approach, CL and LT met regularly to discuss the thematic findings. When there was disagreement, US established agreement on codes. The entire team reviewed and provided comments on the final codebook.

A main goal in this study was to determine if the barriers and facilitators in our safety net setting were similar or different from previously published literature on portal use in other settings—the majority of which have been conducted in integrated health care settings. For this reason, we recoded all the transcripts in a deductive manner to be able to determine if the categories and severity of barriers and facilitators that emerged from our analysis were comparable to previously published work [5,13]. Study staff halted further enrollment in the study after a consensus that thematic saturation had been reached.

Because there was a clear indication of clustering of themes by health literacy status, the transcripts were re-examined by self-reported health literacy status in a secondary exploratory analysis. Although we did not purposively sample for limited health literacy status, we were able to generate some hypotheses for additional types of barriers for patients in this group. We also examined the caregiver transcripts independently from the patient interviews in a similar exploratory fashion.

Finally, we summarized basic usability and accessibility comments as participants looked at screenshots of a hypothetical portal website to understand how they might use the website in the future. This included identifying patients reporting interest in using specific portal features.

## Results

### Enrollment

A total of 45 individuals were approached about the study. Of those approached, 25 (56%) expressed interest in the study, 7 (16%) declined citing lack of interest in computer use, and 13 (29%) declined due to unknown or other reasons (too busy, uninterested in research). Of the 25 who expressed initial interest, 3 subsequently could not be reached, 5 stated they were too busy to schedule an interview, and 1 declined an interview due to emerging health issues. We enrolled 16 participants in the study before reaching thematic saturation.

### Description of Sample

Participants in the study were predominately male (10/16, 63%) and ethnically diverse (50%, 8/16 African American; 19%, 3/16 Latino; 19%, 3/16 Asian or Pacific Islander; 13%, 2/16 white). All patients in the sample were diagnosed with diabetes, 60% (3/5) of caregivers cared for individuals with diabetes and 20% (1/5) of caregivers cared for individuals with multiple chronic conditions, including hypertension, heart disease, chronic kidney disease, and COPD. The mean age of the sample was 56 years (SD 11). More than half (10/16, 63%) of participants had limited health literacy. Overall, participants reported high experience and interest in Internet use: 56% (9/16) expressed high interest in using the Internet to manage their health care and 69% (11/16) were daily users of the Internet. All but 2 participants reported at least occasional Internet use (Table 1).

**Table 1:** Participant demographics.

Characteristic		Overall n=16	Patients n=11	Caregivers n=5
Age (years), mean (SD)		56 (11)	57 (8)	52 (16)
**Gender, n (%)**				
	Male	10 (63)	9 (82)	1 (20)
	Female	6 (38)	2 (18)	4 (80)
**Race/Ethnicity, n (%)**				
	Black or African American	8 (50)	5 (45)	3 (60)
	Hispanic/Latino	3 (19)	3 (27)	0 (0)
	Asian or Pacific Islander	3 (19)	2 (18)	1 (20)
	White or Caucasian	2 (13)	1 (9)	1 (20)
**Role, n (%)**				
	Patient	11 (69)	11 (100)	N/A
	Caregiver	5 (31)	N/A	5 (100)
**Health literacy status, n (%)**				
	Limited	10 (63)	8 (73)	2 (40)
	Adequate	6 (38)	3 (27)	3 (60)
**Interest in using Internet to manage health, n (%)**				
	High	9 (56)	5 (45)	4 (80)
	Some	4 (25)	3 (27)	1 (20)
	None	3 (19)	2 (18)	0 (0)
	Don’t know	1 (6)	1 (9)	0 (0)
**Frequency of Internet use, n (%)**				
	Daily	11 (69)	7 (64)	4 (80)
	Weekly	2 (13)	1 (9)	1 (20)
	Every 2-3 Weeks	1 (6)	1 (9)	0 (0)
	Never	2 (13)	2 (18)	0 (0)
**Internet access, n (%)**				
	Personal computer	13 (81)	9 (82)	4 (80)
	Personal mobile phone	10 (63)	5 (45)	5 (100)
	Computer in public setting	2 (13)	2 (18)	0 (0)

### Major Categories

Overall, the 5 major categories characterizing the barriers and facilitators for portal use were similar to the previously published research on this topic: (1) computer or Internet access, (2) technological skills and interest, (3) security and privacy of information, (4) patient-provider relationship, and (5) chronic illness self-management.

The secondary analysis by health literacy status showed a much higher prevalence of barriers for participants with limited health literacy within several of these categories. These findings are highlighted subsequently.

#### Computer or Internet Access

Overall, the majority of participants reported having consistent and easy access to a personal computer, tablet, or phone. All participants but one owned a computer or mobile device with Internet access. Two participants accessed the Internet solely using a mobile device or tablet, whereas 2 participants with limited health literacy reported accessing a computer or the Internet in public areas, such as a library, classroom, or through a friend:

I go to the library sometimes or a friend’s house or something there, or when I get with the tutor or something and they’re teaching me something, they’ll teach me on their computer or stuff like that.

Male patient, age 56-60 years, Hispanic/Latino, with limited health literacy

In addition, 2 individuals with limited health literacy expressed concerns about the affordability of the Internet, particularly concerning the cost of mobile data:

I don’t have a camera phone. Plus, I don’t have $35.00 that happens to be the monthly fee.

Male patient, age 51-55 years, Asian or Pacific Islander, with limited health literacy

#### Technological Skills and Interest

Although we required some interest in technology in order to enter the study, there was a wide spectrum of technological proficiency, from very limited experience using computers to formal schooling in computer-related fields. Although 3 participants mentioned age as a limiting factor in being able to keep up with the “new generation” of technology use, overall, participants reported using the computer and Internet for a variety of tasks, including communication with friends and family, research, banking, and shopping. Although the majority of participants with adequate health literacy had advanced knowledge of computers, all participants with limited health literacy described their skill levels as being potentially limiting with respect to using a portal:

[My doctor] knows that I’m into computers. I’m a major in computers so [using the portal] is up my alley.

Male patient, age 41-45 years, Asian or Pacific Islander, with adequate health literacy

Yes, [sending an email] would teach me how to type and all that.

Patient, age 46-50 years, African American, with limited health literacy

Although some difficulties reflected issues of cognitive overload, such as difficulty remembering passwords, others represented a lack of basic computer skills. Five participants reported comfort using passwords, but expressed that it was often difficult to remember them, exacerbated by the requirement of websites to change passwords at specific intervals. One participant with limited health literacy also reported difficulty creating passwords, particularly understanding the requirements that websites impose to promote password security, and expressed confusion about the requirement to create your own password:

That’s another thing because you got to have so many words and letters. You know, characters, so how do you distinguish that? I mean you say characters, are they letters?...Where do you get that at? Where do you get the password at?

Male caregiver, age 56-60 years, African American, with limited health literacy

Another participant expressed hesitancy using any websites that require a username and password, preferring to complete transactions such as shopping and banking in-person or over the phone:

Usually when I get to those, I don’t log in...it won’t let me in, I won’t get on it.

Male patient, age 56-60 years, Hispanic/Latino, with limited health literacy

#### Security and Privacy of Information

Most participants expressed concern about their health information being online, although there was nuanced understanding of both the benefits and risks of accessing information online. More specifically, 7 participants noted the vulnerability of online systems to hackers:

You hear so many instances where information has been compromised. I mean, the military can be compromised.

Male patient, age 56-60 years, African American, with limited health literacy

Participants were also concerned about the confidentiality of their health information, particularly sensitive diagnoses and medications. At the same time, 4 participants were unconcerned about security breaches. Participants believed that their personal information was already publicly available through online searches. In addition, they expressed that hackers would find little value in their personal information because they felt unimportant or lacked employment for which the leak of sensitive information would be a threat. Despite concerns about security, 2 participants noted a trust in the ability of a complicated password to improve the security of the hypothetical portal:

I guess for me, more secure is to give a special password. One key. One key to keep it confidential to go in.

Male patient, age 56-60 years, Hispanic/Latino, with limited health literacy

Two participants with limited health literacy described past experiences with computer viruses or information breaches, contributing to their current concerns about online security:

Hackers getting [into] everything...I had to change banks because...they had everything—my name and address—my mom’s maiden name.

Male patient, age 56-60 years, Hispanic/Latino, with limited health literacy

In addition, they described their distrust of potential security measures, including the ability of researchers and industry members to access their health information:

Regardless of what a person says that this site is secured and all that, I just don’t believe it...It’s not only hospitals but pharmaceutical and every researcher will tap into my information. That’s the thing that I worry about.

Male patient, age 51-55 years, Asian or Pacific Islander, with limited health literacy

#### Patient-Provider Relationship

All but one participant noted the benefits of portal use, mainly the option to securely message their provider to get answers to questions not requiring a visit. Five participants also discussed how accessing their personal health records would improve the effectiveness of their in-person visits. Participants with adequate health literacy possessed a more advanced understanding about how improving transparency and knowledge about their health could improve visits by allowing them to ask their providers more specific questions about their diet, exercise, medication, and other management topics:

When you go to a visit, you can ask more specific questions. You can say, “I looked at my labs, and I saw that my A1C was dah, dah, dah.” I know that that represents the fact that I have not been as good with my management as I should be.

Male patient, age 61-65 years, African American, with adequate health literacy

However, 4 participants emphasized the value of in-person communication and did not want online secure messaging to diminish or interfere with ongoing in-person communication with their providers. They expressed concern over technology replacing their health care providers:

I wouldn’t want anything like...I’ve seen those things on television where they got the doctor and you see the doctor on the computer screen and stuff. Is that kind of like what you’re talking about?

Male patient, age 56-60 years, Hispanic/Latino, with limited health literacy

Some participants believed providers would have time to interact with them via the portal, especially through secure messaging. On the other hand, 6 participants, including 5 with limited health literacy, were more skeptical, noting past instances in which they had tried to email their providers to no avail:

Everything is now computer, so yes they would have time. When they’re sitting right there looking at your chart on the computer, that’s their time they email about the patient.

Female caregiver, age 51-55 years, African American, with adequate health literacy

Well, unless I’m missing something very basic, email just doesn’t seem to work...it’s like it goes into this pot.

Male patient, age 61-65 years, African American, with limited health literacy

#### Chronic Illness Self-Management

All participants expressed positive statements about the portal in relation to their health management, including coordination of care and health promotion. For diabetes patients, the option to view past test results was noted as being particularly useful in tracking progress and adjusting health behaviors, such as diet and exercise:

Particularly things like the lab would be good...because then you could not fool yourself. You would have your regular [glucometer] record and your quarterly A1C record to compare so you could see the connection and also give the physician the same ability.

Male patient, age 61-65 years, African American, with adequate health literacy

Ten participants noted that using a portal would save them time in managing their health. In addition, patients felt that using the portal would promote patient-driven communication by improving the ability to seek medical advice in between visits on topics including medication side effects, test results, symptoms, and new treatments seen in the media.

[If] I had a consultation with my pharmacist and they’re telling me of the side effects to watch out with some medications I’m taking...[and] I have one of those side effects, I might discuss it with a doctor on email. That would be really helpful.

Female patient, age 46-50 years, white, with limited health literacy

Three participants with limited health literacy were particularly enthusiastic about the option to check their future appointments online, noting past instances where they had missed appointments because they had forgotten or hadn’t received the proper notification:

Because sometimes they don’t get [the appointment reminder] out on time, so they end up at the last minute sending it out or something, and then [you] find out you had an appointment.

Male patient, age 56-60 years, Hispanic/Latino, with limited health literacy

#### Strong Interest Among Caregivers

Of the 5 caregivers in our sample, 4 were female; 2 were romantic partners, 2 were children of a patient, and 1 was an in-home professional caregiver. All major themes were similar among caregivers and patients, but interest in using the portal seemed stronger among the few caregivers in our sample, primarily because they were already highly engaged in health care management tasks. In the dyad interviews with patients and caregivers, there was no hesitancy among patients to share their personal health records with their caregivers. It was clear that caregivers in this study already played a strong role in communicating with providers and portals would be a logical extension of their role. The majority of caregivers that we interviewed were already informally in contact with a patient’s provider via individual email accounts (ie, not through a portal website) and spoke of their experience positively. Caregivers also noted the importance of their role as interpreters of health information:

I think he would be looking at [the portal] with me but he just doesn’t understand so I would just have to relay the message.

Female caregiver for parent, age 21-25 years, Asian or Pacific Islander, with adequate health literacy

In addition, caregivers described their role as advocates in the care of patients, particularly in advocating that patients not miss out on critical in-person visits as a result of the portal:

I would like [Patient] to go have his visit with the doctor and he loves coming to the doctor and seeing his doctors. Do not take that away.

Female in-home supportive service caregiver, age 51-55 years, African American, with adequate health literacy

Caregivers also discussed the potential for portal use to improve their ability to monitor and promote improvements in health behavior.

To be able to monitor him even better ‘cause now I can go on there, I can look, I can see the results, show him what it’s saying in case he forgets, and let him know, this is what you should do. You need to stop doing this and do this.

Female caregiver for parent, age 56-60 years, African American, with adequate health literacy

Caregivers discussed using the patient portal both independently and in tandem with patients through the patient’s account. As opposed to creating a separate proxy account, one caregiver noted that she would create an account for her parent, which she and a sibling would both use to access the portal:

I’m probably going to make my dad one [portal account] and stuff like that...I’ll teach my brother.

Female caregiver for parent, age 21-25 years, Asian or Pacific Islander, with adequate health literacy

#### Portal Usability

After viewing hypothetical portal screenshots, participants expressed some challenges with the medical terminology and lack of language-appropriate information, but thought the portal layout was otherwise straightforward and comprehensive (Textbox 1).

**Perceptions of hypothetical portal example.**

Weaknesses

1. Difficulty understanding portal content

Yes, and then the lab result, even though I won’t understand most of it.

Male patient, age 41-45 years, Asian or Pacific Islander, with adequate health literacy

Probably to see a blood test result. I wouldn’t really—unless somebody explained it, I wouldn’t know what I was looking at, really. It’s like diagnosing your car, tells you all this stuff but then you don’t know what it is. I got so much stuff.

Male patient, age 56-60 years, Hispanic/Latino, with limited health literacy

2. Language access or limited English proficiency

Is there any other options like other languages that you can kind of change the message to? Like not permanently but let’s just say that day, if I teach my dad how to go online and he can look up for himself, like that day when he go on, can he click a certain button that’s not that hard for him to change it, let’s say to Vietnamese.

Female caregiver for parent, age 21-25 years, Asian or Pacific Islander, with adequate health literacy

To be honest with you, unless it’s something interesting to go into that health thing, then I would go. For example, if that’s in my language, I would go.

Male patient, age 51-55 years, Asian or Pacific Islander, with limited health literacy

Strengths

1. Hypothetical portal simple and clear

Yes. It’s much easier than when I was in school. That portal was awful.

Male patient, age 41-45 years, Asian or Pacific Islander, with adequate health literacy

Well it seems really self-explanatory. It’s like really basic, just all right there. I can’t think of anything to add to it.

Female patient, age 46-50 years, white, with limited health literacy

#### Interest in Portal Use

After seeing the example screenshots of the future patient portal, 88% (14/16) of participants reported a willingness to use the future portal website to manage their health care. Looking at specific features, there was highest interest in accessing laboratory results (81%, 13/16), appointments (81%, 13/16), and visit summaries (81%, 13/16).

## Discussion

### Principal Findings

Among a diverse group of patients and caregivers in a safety net clinic, we identified significant barriers to portal use, including concerns about security and privacy, limited technological proficiency, and a desire to preserve in-person aspects of existing patient-provider relationships—most often among patients with limited health literacy. Recruiting only those who expressed at least some interest in using the Internet for health management, our findings are likely conservative in that they represent some of the more engaged patients within our safety net health care system. The majority of participants in our study were African American and male, characteristics which have both been associated with lower portal use [13,14,17,26,36]. Nevertheless, it is important to note there was overall enthusiasm among these participants about the potential of a patient portal to improve aspects of health monitoring, patient-provider relationships, and caregiver burden. This is consistent with interest [21,26,27] and benefits described among low-income patients in past studies [5,21,27].

The overall categories of barriers to portal use in our study were consistent with previous studies: concerns about security [5,37], difficulty understanding medical information [5,21], the desire to preserve verbal communication and in-person contact [5,20,38], and the burden of portal use on clinician workloads [5]. However, because our sample included predominantly patients with limited health literacy, our findings uncovered more pronounced aspects of these barriers in safety net settings, such as access to computers in public settings; negative past experiences with technology, including security breaches and viruses; and a lack of more rudimentary computer skills. In particular, a distrust of potential security measures to prevent access of personal health information by hackers, researchers, and others unauthorized by the patient may hinder patient portal use among safety net patients. This is consistent with past studies indicating that individuals with limited health literacy are less likely to sign onto a portal website [14,39], use portal messaging functions [39], identify blood sugar test results as out of range [19], and contact a provider to discuss abnormal test results from an online portal [19]. Public computer use coupled with relatively widespread security concerns may be particularly relevant to address among patients with lower socioeconomic status, especially because previous studies have suggested that only a minority of a general patient population express hesitancy to use portals because of security [37,40]. Past research has shown that older, low-income patients desire assistance in interpreting their personal health information [41]. Our findings show that health literacy is a major barrier among younger populations within the safety net as well.

Although our sample of caregivers was small (limiting our ability to make strong inference), our findings also imply that there is a potential supportive role for caregivers to facilitate portal use in a safety net setting. Particularly for patients lacking adequate health literacy, English proficiency, and/or the technological know-how or interest to access and interpret their personal health information, there is potential for caregivers to use patient portals to improve their ability to interpret health information, coordinate care, and assist with medical decision making [22]. Past studies have found high interest among patients in sharing their personal health information with caregivers [42,43] and among caregivers in accessing patient health information through health technology such as portals [44,45]. Caregivers in our study expanded on the utility of having access to patient health information to describe a deeper role in caregiving, noting their role as interpreters of this information, guides in decision making and behavior change, and advocates in ensuring quality of care. Our findings illustrate the need for safety net health systems implementing patient portals to address caregiver needs—including strategies for formal proxy processes for patients to officially grant others access to their portal account as well as awareness of informal sharing of username/passwords between family members that is also likely to occur. Because the caregivers in our sample were already highly involved in health care management tasks, further research is needed to understand perspectives on the levels of caregiver access to personal health information [40,42].

### Limitations

Because our small study examined the in-depth perspectives of patients receiving care from one urban safety net hospital, our findings are likely not generalizable to patients receiving care from larger health systems or networks. Furthermore, because our eligibility criteria required participants to speak English and express at least some interest in using the Internet to manage their health, our findings may not be generalizable to those with limited English proficiency or needing even more basic computer or technology training. Finally, our study did not incorporate perspectives on the actual usability of a live portal website. Instead, we focused on gaining in-depth information about the barriers and facilitators to portal use in advance of its rollout to support a patient-centered approach to implementing our portal system-wide within the San Francisco Health Network.

### Conclusions

Our findings indicate that interest in using patient portals may not always match the technological proficiency of more vulnerable patients. This indicates a need for safety net health systems or other social service providers (eg, library, adult literacy classes) to provide training not only in portal use, but also in equipping patients with the basic computer and health literacy to effectively use a portal. To address patient concerns, it is important for health care systems implementing portals to assess the potential effects of the replacement of in-person or verbal communication resulting from portal use and establish high levels of online security.

From a national perspective, our findings suggest that widespread EHR and portal implementation may be hindered by patient engagement challenges in the coming years, especially with respect to health literacy and language proficiency status. Although incentives to promote meaningful use have been successful at driving health systems to implement health information technology, these standards do not guarantee that safety net health systems will adopt the newest or most accessible technologies on the market [46,47] or that patient portals will be accessible or useful to all patients, especially those who may face additional limitations in literacy and technology experience. Addressing health literacy and other barriers may best be achieved through patient-centered approaches to the adoption of health information technology at the planning, implementation, and evaluation stages [48]. If implemented with patient perspectives in mind, patient portals have the potential to be a convenient and effective way to improve self-management and quality of care for patients and caregivers receiving care from safety net settings.

## Appendix

Multimedia Appendix 1 Interview Guide.

## Abbreviations

COPD: chronic obstructive pulmonary disease
EHR: electronic health record
GMC: General Medicine Clinic
HITECH: Health Information Technology for Economic and Clinical Health
SFGH: San Francisco General Hospital
