# Supplementary material for: Barriers and Facilitators to Online Portal Use Among Patients and Caregivers in a Safety Net Health Care System: A Qualitative Study
Source: J Med Internet Res. 2015 Dec 3;17(12):e275. doi: 10.2196/jmir.4847 (PMC4704882; doi:10.2196/jmir.4847)
Supplement: Multimedia Appendix 1 [file jmir_v17i12e275_app1.pdf]

Multimedia Appendix 1. Interview Guide

In-Depth Interviews About Online Portals – Discussion Guide

Today we're going to talk about you/your family's health and how you manage you/your family's health, including how you use technology in your everyday life. I appreciate your honesty in sharing your experiences.

1. Current Health Situation (15 minutes)

a) How is your health these days?
b) How active would you say you are in managing your own health?
c) Are there specific things you are doing to try to stay healthy?
d) How do you get the facts you need about your health? Your chronic conditions?
e) Are there any other types of information or people that you trust about your health?
f) Do you have a family member or a friend who helps you with your health?

2. Healthcare (15 minutes)

a) Do you have a regular doctor?
b) Tell me about what it was like at your most recent visit – how did that go?
c) What has your interaction been with other people in the clinic like nurses?
d) What happens if you have a question for your doctor in between your office visits?
e) How do you feel about calling your doctor in between visits?

A lot of people like to get questions about their health answered on the Internet, so now we are going to shift and talk about that for a minute.

3. Technology (25 minutes)

a) Do you have a computer? Mobile phone?
b) How do you use the Internet?
c) (If applicable) Which device do you use to go online *most* often?
d) A lot of people say they need their son and daughter to get them on the Internet, or that they can only do a few things on their own. How about you?
e) What kinds of things do you do online?
f) Tell me about what a typical week looks like in terms of using the Internet.
g) Some of these sites require a log-in and password. Tell me your thoughts about that.

h) We talked about health information earlier. Do you use the Internet to look up things about your health or the health of someone in your family?

i) Some doctors and nurses are now emailing with patients or providing medical information to patients via the Internet. How do you feel about that for you personally?

j) How would you feel about seeing your test results online?

k) How would you feel about viewing your medical history online?

l) How would you feel about refilling your medications online?

m) How would you feel about scheduling an appointment online?

n) The reason I am doing this study is because SFGH is developing a website called an online portal that will have access to patients' medical information and allow emailing with doctors and other providers. I am trying to understand how we can improve this and make it the best for our patients. (Show screenshots.) What are your thoughts about this?

o) Which of the following portal features would you use?

   a. The portal overall
   b. Lab results
   c. Secure messaging
   d. Appointment list
   e. Visit summary
   f. Prescription refill
   g. Other

p) If your doctor or someone else in the clinic recommended this website, how would you feel about that?

q) What else about this online portal website would be useful to you?

r) Do you think that using an online portal website could replace an in-person visit with you doctor?

s) How would you feel if this website had information about general health or diabetes topics, like videos for healthy eating or exercises to do at home?

4. Closing (5 minutes) – I am happy to answer any of your questions that we didn't get to earlier.

a) Do you have any other questions?
